# Seed-specific elevation of non-symbiotic hemoglobin *AtHb1*: beneficial effects and underlying molecular networks in *Arabidopsis thaliana*

**DOI:** 10.1186/1471-2229-11-48

**Published:** 2011-03-15

**Authors:** Johannes Thiel, Hardy Rolletschek, Svetlana Friedel, John E Lunn, Thuy H Nguyen, Regina Feil, Henning Tschiersch, Martin Müller, Ljudmilla Borisjuk

**Affiliations:** 1 Leibniz-Institut für Pflanzengenetik und Kulturpflanzenforschung (IPK), Corrensstr. 3, 06466 Gatersleben, Germany; 2 Max Planck Institute of Molecular Plant Physiology, Science Park Golm, 14476 Potsdam-Golm, Germany; 3 Virus Surveillance and Diagnostic Branch, Influenza Division/NCIRD, Centers for Disease Control and Prevention, 1600 Clifton Rd, Mail Stop G-16, Atlanta, GA 30333, USA

## Abstract

**Background:**

Seed metabolism is dynamically adjusted to oxygen availability. Processes underlying this auto-regulatory mechanism control the metabolic efficiency under changing environmental conditions/stress and thus, are of relevance for biotechnology. Non-symbiotic hemoglobins have been shown to be involved in scavenging of nitric oxide (NO) molecules, which play a key role in oxygen sensing/balancing in plants and animals. Steady state levels of NO are suggested to act as an integrator of energy and carbon metabolism and subsequently, influence energy-demanding growth processes in plants.

**Results:**

We aimed to manipulate oxygen stress perception in *Arabidopsis *seeds by overexpression of the non-symbiotic hemoglobin *AtHb1 *under the control of the seed-specific LeB4 promoter. Seeds of transgenic *AtHb1 *plants did not accumulate NO under transient hypoxic stress treatment, showed higher respiratory activity and energy status compared to the wild type. Global transcript profiling of seeds/siliques from wild type and transgenic plants under transient hypoxic and standard conditions using Affymetrix ATH1 chips revealed a rearrangement of transcriptional networks by *AtHb1 *overexpression under non-stress conditions, which included the induction of transcripts related to ABA synthesis and signaling, receptor-like kinase- and MAP kinase-mediated signaling pathways, WRKY transcription factors and ROS metabolism. Overexpression of *AtHb1 *shifted seed metabolism to an energy-saving mode with the most prominent alterations occurring in cell wall metabolism. In combination with metabolite and physiological measurements, these data demonstrate that *AtHb1 *overexpression improves oxidative stress tolerance compared to the wild type where a strong transcriptional and metabolic reconfiguration was observed in the hypoxic response.

**Conclusions:**

*AtHb1 *overexpression mediates a pre-adaptation to hypoxic stress. Under transient stress conditions transgenic seeds were able to keep low levels of endogenous NO and to maintain a high energy status, in contrast to wild type. Higher weight of mature transgenic seeds demonstrated the beneficial effects of seed-specific overexpression of *AtHb1*.

## Background

Hemoglobins (Hbs) represent a large ubiquitous group of proteins found in all kingdoms of life [1]. In plants, there are three major groups: (i) symbiotic or leghemoglobins, facilitating oxygen diffusion to nitrogen-fixing bacteria in nodules of plants (ii) non-symbiotic hemoglobins (nsHbs) found in numerous species, and (iii) the poorly characterized group of truncated hemoglobins [2,3]. The nsHbs in turn are divided into class-1 (Hb1) and class-2 (Hb2) subgroups based on phylogenetic analyses and structural/kinetic properties of the proteins. Hb1 has a superior affinity for oxygen and its expression is induced during hypoxic stress [4,5]. Notably, its overexpression in plants was shown to enable the cell to maintain high ATP levels under hypoxia [6]. This finding was later explained by the ability of Hb1 to detoxify reactive nitrogen species like nitric oxide (NO) [7,8]. NO is a key signaling molecule involved in multiple processes, like stomatal closure, programmed cell death and pathogen resistance [9]. The level of NO rises under hypoxia, and is related to the availability of nitrite [4,5,10]. Despite the clear effects of Hb1 on the abundance of NO, the *in vivo *sources of NO, its targets as well as signaling mechanisms are still a matter of debate [11].

Seeds of crop species experience a regular oxygen deficiency during both development and germination [12]. This leads to ATP limitation and subsequently, to a restriction of high energy-demanding processes like cell division, growth and storage product synthesis [13]. Oxygen limitation is in part caused by the high diffusional impedance of certain seed structures. Thus, even the tiny seeds of *Arabidopsis thaliana *operate close to the edge of hypoxia. Consequently, a moderate decrease in atmospheric oxygen concentration to about half saturation already induces clear metabolic restrictions in *Arabidopsis *seeds [14]. The molecular mechanisms of the seeds' response to hypoxia might resemble those of other plant organs [15-17] and tissue types [18] of *Arabidopsis*, but detailed transcriptomic studies are lacking.

Based on a series of *in vitro *experiments, we recently proposed that the steady state level of NO in seeds acts to integrate carbon and energy metabolism [5]. Upon application of either NO scavengers or NO inducing compounds, seeds responded with alterations in both oxygen uptake and metabolic activity evident at both the transcript and metabolite level. Congruently, respiratory activity of isolated seed mitochondria showed clear responses to NO/nitrite [10]. However, the extent to which such *in vitro *studies mirror the *in vivo *situation can always be questioned. Here, we used the non-symbiotic hemoglobin *AtHb1 *to manipulate endogenous levels of NO in seeds. The *AtHb1 *(also referred to as *AtGLB1 *or *AHb1 *in the literature) was overexpressed under the control of the seed-specific LeB4 promoter in *Arabidopsis thaliana*. Comparative analyses of both transcripts and metabolites were performed with wild type (WT) and transgenic plants grown under standard conditions as well as under moderate hypoxic stress treatment. Results indicate that *AtHb1 *overexpression led to several alterations in transcriptional and metabolic networks, resulting in improved seed yield (weight).

## Results

### Overexpression of *AtHb1 *is targeted to seed and increases seed weight

**Figure 1:**
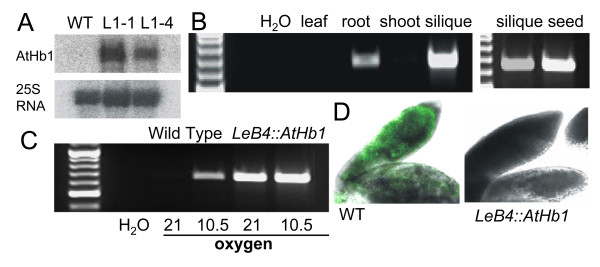
**Effects of *AtHb1 *overexpression in *Arabidopsis *seeds**. (A) Northern blot analysis of *AtHb1 *expression in WT and homozygous transgenic plants (L1-1 and L1-4) at 45 DAG, 25S RNA was used as loading control. (B) RT-PCR analysis of *AtHb1 *expression in different tissues of L1-1. (C) RT-PCR analysis of *AtHb1 *expression in siliques of WT and L1-1 under control conditions and moderate hypoxia. (D) Fluorescence detection assay of NO using DAF-2DA. Fluorescence signals (green) indicate NO accumulation.

We generated transgenic *Arabidopsis *plants expressing the endogenous *AtHb1 *under the control of the seed-specific LeB4 promoter [19]. Northern blot analysis of siliques from homozygous T3 plants demonstrated significant *AtHb1 *expression, whereas in WT plants the endogenous *AtHb1 *expression was not detectable under standard conditions (Figure 1A; for additional transgenic lines see below). RT-PCR analysis showed that, overexpression of *AtHb1 *under the control of the LeB4 promoter was restricted to siliques/seeds in the transgenic plants (minor expression in roots; Figure 1B). Comparison of manually isolated seeds with whole siliques (including seeds) revealed that LeB4-driven expression is mainly localized in seeds in agreement with previous results [19]. To avoid any stress-induced artefacts that might be induced by dissection of seeds from the siliques, whole siliques were used for further studies

*AtHb1 *overexpression did not alter the vegetative growth of transgenic plants. Also timing of developmental programmes, like induction of flowering and silique development were not affected by transgene expression. Interestingly, mature seeds of transgenic plants revealed a higher weight (Table 1) whereas seed number and composition were unaffected.

**Table 1:** Characteristics of mature seeds of WT and *AtHb1-*overexpressing lines

	WT	Line 1-1	Line 1-4
Total lipid (% DW)	34.8 ± 3.0	36.2 ± 6.5	29.4 ± 10.2
Total protein^1 ^(% DW)	22.6 ± 2.0	21.4 ± 0.6	23.0 ± 1.3
Total carbon (% DW)	53.1 ± 1.4	54.9 ± 1.4	53.7 ± 1.2
Seed weight^2 ^(μg)	**17.8 ± 3.5**	**23.0 ± 3.2**	**21.1 ± 2.3**
% increase in seed weight	**100**	**131 ± 18**	**130 ± 15**
Seed number per plant^3^	13231 ± 2576	16851 ± 4685	15115 ± 2273

Data are means (± SD). Bold values indicate statistically significant differences (t-test, p < 0.05).

^1 ^calculated from total N content * 6.25

^2 ^analysed in three generations (T3-T5)

^3 ^calculated from seeds per pod * pods per plant

### Overexpression of *AtHb1 *reduces the endogenous level of nitric oxide in seeds

A qualitative fluorescence assay with diaminofluoresceine-2-diacetate (DAF-2DA) was used for detection of endogenous NO in WT and *AtHb1 *embryos under standard and hypoxic stress conditions.

**Figure 2:**
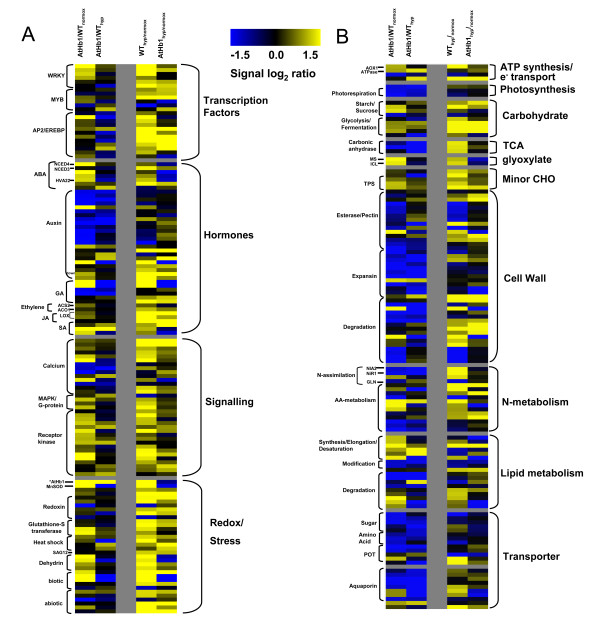
**Heat map display of differentially expressed genes involved in regulation/redox processes and primary metabolism**. Columns indicate mean signal log2 ratios of differentially expressed genes in at least one comparison. Each comparison is arranged into vertical columns in the following order: column 1, *AtHb1 *overexpression versus WT under control conditions; column 2, comparison of both genotypes under hypoxic conditions; column 3, WT under hypoxia versus WT under control conditions; column 4, *AtHb1 *under hypoxia versus *AtHb1 *under control conditions. Blue indicates downregulation, yellow indicates upregulation. Genes organized by pathways, (A) regulation/signaling and stress response, (B) primary metabolism and transport. Additional file 4 contains the gene lists used.

To induce moderate hypoxic stress in the seeds, intact plants were treated with artificial air mixes containing only 10.5 kPa oxygen (corresponding to half atmospheric oxygen saturation) for one hour. Seeds of WT plants showed a slight induction of *AtHb1 *expression under these conditions (Figure 1C), but its expression level was still much lower than in the transgenic plants. Microarray results confirmed the higher abundance of *AtHb1 *mRNA in transgenics under hypoxia (>3-fold, Figure 2A, marked by asterisk).

Under standard growth conditions, NO was not detectable in the embryos of either WT or *AtHb1 *plants using the fluorescence assay. Possibly, the steady state level of NO was below the detection limit of the assay. Under moderate hypoxia, WT showed a clear fluorescence signal (in green), while *AtHb1 *overexpressors did not (Figure 1D). This indicated strongly decreased NO levels in the latter. Thus, the transgenic approach resulted in lower levels of NO. The induction of *AtHb1 *expression (Figure 1C) and enhanced NO emission (Figure 1D) in WT further indicated that the moderate stress treatment was sufficient to induce hypoxia in seeds.

### Experimental set up for microarray analysis

To assess changes in gene expression in seeds/siliques due to *AtHb1 *overexpression in detail, we focused on line L1-1, which showed the strongest transgene expression. Six other independent transgenic lines were involved in further studies (see below).

WT and transgenic plants were exposed to moderate hypoxia (10.5 kPa) or normoxia (21 kPa; control) for one hour. Three biological replicates were used for hybridization to Affymetrix ATH1 arrays. A cluster dendrogram of transcript signal intensities from the 12 arrays showed a high reproducibility of the biological replicates from each data set (genotype+treatment), and indicated a greater influence of the genotype than the treatment on transcriptional profiles (Additional file 1A). Transcript analysis by qRT-PCR showed a high correlation (R^2 ^= 0.83) with the microarray data, confirming the reliability of the data (Additional file 1B).

We compared the transcriptome of WT and *AtHb1 *siliques/seeds under control and hypoxic conditions, as well as the hypoxic responses in each genotype. Differentially expressed genes were extracted from the data base by applying the following cutoffs: a fold-change of >2 and a p-value of <0.05. A total of 1,010 genes were identified as differentially expressed in all of the comparisons. Differentially expressed genes were grouped into eight clusters (Additional file 2 and 3), classified into functional groups using the MapMan bin code [20] and ordered by pathways. The heat map display in Figure 2 gives a detailed view of the altered pathways (also listed in Additional file 4).

**Figure 3:**
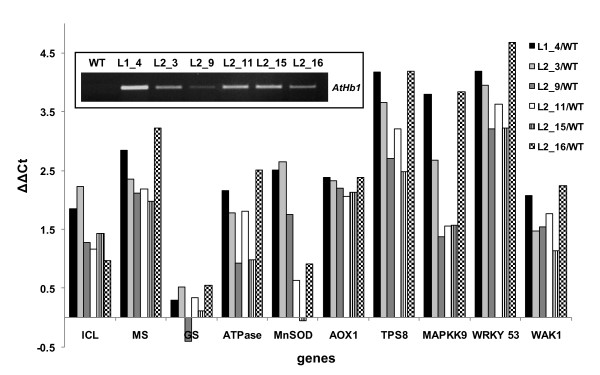
**Transcript ratios of *AtHb1*-induced marker genes in different *AtHb1*-overexpressing lines relative to WT**. *AtHb1 *transcript accumulation in siliques of different transgenic lines obtained by RT-PCR is depicted in the inset. For transcript analysis siliques of 45 DAG plants have been used. qRT-PCR analysis was conducted for genes showing a preferential expression in *AtHb1 *(Line 1-1) compared to WT under control conditions as measured by microarray analysis. MnSOD (At3g56350), ICL (At3g21720), MS (At5g03860), AOX1 (At1g32350), WAK1 (At1g21250), GS (At5g53460.), ATPase (Chl) (At1g15700 ), TPS8 (At1g70290), MAPKK9 (At1g73500), WRKY 53 (At4g23810).

To confirm that microarray data of L1-1 are reproducible in further transgenic lines, we analyzed the expression of selected genes in six other *AtHb1*-overexpressing lines by qRT-PCR (Figure 3). A set of transcripts that have been shown in the microarray analysis to be upregulated by *AtHb1 *overexpression was selected for qRT-PCR analysis. All of the transgenic lines exhibited an enhanced expression of the genes from representative signaling, redox and metabolic pathways compared to the WT, indicating similar expression profiles due to *AtHb1 *overexpression in independent transgenic lines.

### *AtHb1 *overexpression induces stress-related regulatory pathways under non-stress conditions

Comparison of the transcriptome of WT and *AtHb1 *overexpressors under control conditions revealed multiple changes (Table 2). The effects on molecular networks involved in stress responses and signaling were particularly pronounced (Figure 2A). WRKY and AP2/EREBP transcription factors, as well as genes related to hormone metabolism, i.e. abscisic acid (ABA), salicylic acid (SA) and jasmonic acid (JA), were found to be upregulated in *AtHb1 *seeds. Moreover, many genes involved in signaling processes, like MAPK kinases and receptor kinases, and in redox/stress-related processes were strongly induced. This trend was also confirmed by analysis of differentially expressed genes for indicative over- and underrepresented gene ontology categories (GO terms). Upregulated genes in *AtHb1*-overexpressing plants showed a strong enrichment of GO categories involved in stress responses (Additional file 5).

**Table 2:** Number of differentially expressed genes

Number of genes	*AtHb1*_control vs. WT_control	*AtHb1*_hyp vs. WT_hyp	WT_hyp vs. WT_control	*AtHb1*_hyp vs. *AHb1*_control
upregulated	270	176	351	153
downregulated	205	197	62	101

Genes with log_2 _signal ratios > 1 and p-values < 0.05 between WT and *AtHb1*-overexpressing plants under control and hypoxic conditions and after hypoxic treatment of each genotype were extracted from the data base.

Among transcription factors, four transcripts, encoding WRKY 33, 40, 53 and 75, were significantly upregulated. *WRKY *genes have been shown to play a role in hypoxic responses of different cell types of *Arabidopsis *[18]. Prominent differences in hormone metabolism were observed for ABA, SA and auxin-related genes. A strong upregulation of *NCED4 *was accompanied by preferential expression of transcripts encoding ABA-responsive proteins (At2g40170, At3g02480, At5g62490). The elevation of transcripts involved in ABA metabolism/signaling is consistent with an overrepresentation of ABRE binding sites in the 5'-flanking regions of *AtHb1 *coexpressed genes (Table 3). Auxin transport and signaling is commonly downregulated in transgenics. Fourteen genes, among them auxin transporter (*AUX1*), auxin-induced genes (*GH3, SAUR, IAA, ARF1*) were strongly downregulated, whereas two transcripts encoding auxin downregulated protein ARG10 were upregulated.

**Table 3:** Promoter motifs of differentially expressed genes

Motif (1000 bp upstream)	p-value	Motif (1000 bp upstream)	p-value
***AtHb1 *vs. WT upregulated control**		***AtHb1 *vs. WT downregulated control**	
ABRE-like binding site motif	< 10e-10	MYCATERD1	< 10e-5
ABRE binding site motif	< 10e-5	AtMYC2 BS in RD22	< 10e-5
ACGT ABRE motif A2OSEM	< 10e-10		
ABREATRD22	< 10e-5		
GADOWNAT	< 10e-10		
Ibox promoter motif	< 10e-5		
Z-box promoter motif	< 10e-10		
CACGTG motif	< 10e-10		

***AtHb1 *vs WT upregulated hypoxia**		***AtHb1 *vs WT downregulated hypoxia**	
no enrichment		MYCATERD1	< 10e-7
		AtMYC2 BS in RD22	< 10e-7
		RY-repeat promoter motif	< 10e-6

**WT hyp vs WT control upregulated**		**WT hyp vs WT control downregulated**	
W-box/WRKY	< 10e-5	no enrichment	
I-Box	< 10e-7		
ABRE-like binding site motif	< 10e-9		
ABRE binding site motif	< 10e-7		
ACGT ABRE motif A2OSEM	< 10e-10		
DRE core motif	< 10e-8		
DREB1A/CBF3	< 10e-6		
CACGTG motif	< 10e-10		
GADOWNAT	< 10e-10		
AtMYC2 BS in RD22	< 10e-5		
MYCATERD1	< 10e-5		
Z-box promoter motif	< 10e-7		
EveningElement promoter motif	< 10e-5		

***AtHb1 *hyp vs *AtHb1 *control upregulated**		***AtHb1 *hyp vs *AtHb1 *control downregulated**	
EveningElement promoter motif	< 10e-5	ABRE-like binding site motif	< 10e-7
		ABRE binding site motif	< 10e-5
		ACGT ABRE motif A2OSEM	< 10e-9
		G-box LERBC	< 10e-5
		GADOWNAT	< 10e-9
		RY-repeat promoter motif	< 10e-6

Overrepresented motifs with p-values < 10e-4 were selected for comparative analysis.

Genes implicated in signaling pathways, like receptor kinases, *wall-associated kinase 1 *(*WAK1*, At1g21250) and *MAPK kinase 9 *(At1g73500) were also upregulated compared to WT. WAK1 is a transmembrane protein containing a cytoplasmic Ser/Thr kinase domain and an extracellular domain bound to the pectin fraction of cell walls [21], thus enabling communication between cell wall and cytoplasm. Phosphorylation via WAKs has been shown to play a pivotal role in cell wall metabolism [22], which was significantly altered by *AtHb1 *overexpression. *WAK1 *expression is induced by SA treatment [23], thus, higher expression of *WAK1 *and two S-adenosyl-L-methionine:carboxyl methyltransferases indicates an involvement of SA signaling in the regulatory networks controlled by *AtHb1*. In addition, the expression of 11 transcripts encoding receptor kinases, such as transmembrane kinase RLK5 and other leucine-rich repeat family proteins as well as Ser/Thr kinases, revealed the presence of different signaling pathways. Interestingly, RLK7 (At1g09970) has recently been shown to be involved in the control of seed germination and tolerance to oxidative stress [24]. Using genetic approaches the authors provided evidence for a positive correlation of RLK7 expression and enhanced tolerance against H2O2.

Transcripts encoding proteins involved in redox homeostasis, such as manganese superoxide dismutase (MnSOD, At3g56350) and two glutathione-S-transferases, were upregulated in *AtHb1 *overexpressors. This was accompanied by higher expression of defence-related proteins, i.e. dehydrins and major latex proteins (MLP-related) (Figure 2A).

Ubiquitin-mediated proteolysis is essential for plant development and responses to environmental stimuli [25]. *AtHb1 *induced the expression of three RING finger E3 ligases of the C3CH4-type (At4g14365, At2g27940, At1g30860) and two F-box proteins (SKP1/At2g45950 and kelch repeat/At1g80440) (Additional file 6). RING finger ligases and E3 ligases from the SKp1, F-box (SCF) complex play an essential role in auxin metabolism by degrading AUX/IAA proteins, and thereby regulating concentrations of IAA [25]. This is probably linked to downregulation of auxin transport and signaling in *AtHb1 *plants.

### *AtHb1 *overexpression in seeds alters expression of genes involved in primary metabolism

*AtHb1 *overexpression induces various changes in transcripts related to carbohydrate, cell wall, N- and lipid metabolism, as well as potentially associated transporter gene activities and photosynthesis. As deduced from GO analysis of transcript data, the cell wall was the most affected cellular compartment in *AtHb1 *seeds showing a clear underrepresentation (Additional file 5). Other decreased biological processes are linked to cell wall biogenesis and modification. This is illustrated by the concurrent downregulation of more than 30 cell wall-related genes encoding cellulose synthases, arabinogalactan-proteins (AGPs), pectinesterases, expansins, xyloglucan-xyloglucosyl transferases and polygalacturonases (see MapMan visualization, Additional file 7). This indicates a strong repression of cell wall synthesis, cell wall modification, pectin degradation, cell expansion and cell wall turnover. Two transcripts (At1g70290, At2g18700) encoding class II trehalose-6-P synthase/phosphatase (TPS8, TPS11) were preferentially expressed in *AtHb1 *plants. These transcripts are also potentially linked to cell wall metabolism, as it was found that perturbation of trehalose metabolism in embryos of the *tps1 *mutant leads to changes in cell wall composition and thickness [26]. Lipid metabolism also showed transcriptional alterations; fatty acid elongation and desaturation were activated but transcripts involved in squalene and steroid metabolism were repressed. In addition, transcripts for malate synthase and isocitrate lyase (key enzymes of the glyoxylate pathway) were upregulated in *AtHb1 *seeds. Furthermore, transcripts encoding the 4Fe-4S cluster protein of photosystem I and key enzymes of the photorespiratory pathway (glycolate oxidase/GOX, At3g14415; serine hydroxymethyltransferase 4/SHMT4, At4g13890) were downregulated.

Nitrogen metabolism appears to be affected in *AtHb1 *seeds based on the downregulation of *nitrate reductase 2 *(*NIA2*, At1g37130) and *nitrite reductase 1 *(*NiR1*, At2g15620). Several transcripts involved in amino acid metabolism differed significantly between transgenic and WT (S-adenosylmethionine synthetase, S-adenosyl-L-homocysteinase, asparaginase, cystine lyase, delta-1-pyrroline-5-carboxylate synthetase).

Several transporter gene activities were commonly downregulated in *AtHb1 *seeds, namely those involved in sugar, amino acid and oligopeptide transport (POT). Most of these are proton-coupled transporters. In addition, five genes from different subgroups of the aquaporin family were downregulated. These genes play a role in nutrient flow and/or are implicated in remobilization [27,28].

### Changed gene interactions due to *AtHb1 *overexpression point to alterations in cell wall metabolism

To infer gene-to-gene interactions we used the MRNET approach which extracts statistical dependencies between genes [29]. The reconstructed network of gene interference for the top 20 genes that are differentially expressed between WT and *AtHb1 *overexpressing seeds under control conditions showed clear differences (Additional file 8). In WT, the gene encoding fasciclin-like arabinogalactan protein 13 (FLA13; At5g44130) was the central hub. AGPs, such as FLA13, play a role in plant cell elongation/cell wall biogenesis, and are assumed to act as signal molecules [30]. Proteins containing fasciclin domains have also been shown to function as adhesion molecules in a broad spectrum of organisms [31]. There were multiple interactions of this hub with genes encoding proteins localized to the cell wall (e.g. xyloglucan:xyloglucosyl transferase, xyloglucan endotransglycosylase 3 (XTR3), proline-rich protein 2 (ATPRP2) and acid phosphatase class B family protein) or otherwise involved in extracellular matrix modifications (e.g. midchain alkane hydroxylase, which is involved in cuticular wax biosynthesis; [32]). Most of the genes are implicated in stress-responses and related to hormone (ABA, GA) action. Overexpression of *AtHb1 *directly or indirectly perturbed the strong multiple interactions of the hub gene *FLA13*, shifting the main regulatory point to *ATPRP2*. It has been shown, that *ATPRP2 *is one of the key genes involved in cell specification [33]. Cell specification in the embryo might be coupled to maturation processes, which are characterized by high storage- but extremely low mitotic-activity. Downregulated expression of *ATPRP2 *(and associated genes) in *AtHb1 *plants might therefore indicate decelerated cell specification and thus, an extented growth phase.

### Evaluation of adaptive stress responses in wild type seeds

Most of the adaptive responses in WT seeds have also been described for shoots and roots of *Arabidopsis *plants. Mustroph et al. [18] identified a core set of 49 translated hypoxia-induced mRNAs in 21 different *Arabidopsis *cell populations. From this core set, 35 genes (~70%) were also found to be upregulated in seeds, indicating similar adaptation strategies to hypoxia regardless of tissue/organ identity. The possible induction of the glyoxylate cycle in combination with lipid degradation (phospholipase C, phosphodiesterase) was not observed in other *Arabidopsis *tissues and might therefore be seed-specific. The induction of the glyoxylate cycle could represent an alternative mechanism to generate sugars and sustain energy supply under unfavourable conditions in seeds. Interestingly, malate synthase and isocitrate lyase are also enhanced in carbon-starved cucumber cotyledons [34]. The higher expression of genes involved in sugar, amino acid, oligopeptide and general nutrient (aquaporins) transport in WT (column 2 in Figure 2B) and the significantly reduced sucrose concentrations (see below) indicates nutrient, particularly sugar, depletion in WT upon hypoxia.

In general, WT seeds showed a strong transcriptional and metabolic response to moderate hypoxia. Metabolism and signaling of hormones (ABA, ethylene, JA, SA and GA) which are described to be important triggers in response to oxidative stress [15,16] are strongly induced in seeds. Activation of specific transcription factors and signaling pathways nicely illustrates a cross-talk of hormone action and regulatory pathways, particular for ethylene. Upregulation of *MAPKK9, MAPK3 *(At3g45640) accompanied by activation of *ACC oxidase1 *(At2g19590) as well as ten members of the AP2/EREBP family represents an example how signaling cascades are linked together in adaptive stress responses. Experiments with maize suspension cultures showed a correlation of varying class-1 hemoglobin levels and changed NO concentrations with ethylene formation [35]. Enhanced ethylene biosynthesis under hypoxia is linked to lower hemoglobin expression, coinciding with the stronger induction of ethylene synthesis and signaling in the WT compared to the *AtHb1 *plants in our experiments. Beside the strong activation of several WRKY transcription factors and *MYB44 *(At5g67300), transcripts related to redox regulation were clearly induced. Rising concentrations of H2O2 in WT upon hypoxia correlate with transcriptional activation of several ROS generating/scavenging enzymes coinciding with other studies [36,37]. The upregulation of several class II TPS genes and the reduction of trehalose-6-P (T6P) levels was part of the hypoxic response in WT (two of them are also induced in transgenics under control conditions). Interestingly, T6P metabolism was identified as being part of a hypoxic response that is conserved in some pro- and eukaryotes [38]. T6P may be involved in coordination of carbon partitioning between primary metabolism and cell wall synthesis [39]. Therefore, altered expression of TPS genes - together with changes in cell wall metabolism - accentuates the possible role of T6P metabolism in regulation of carbon partitioning. In general, the alterations in regulatory and metabolite pathways provide a framework of seed-specific responses to hypoxia.

### *AtHb1 *overexpression attenuates transcriptional stress responses

**Figure 4:**
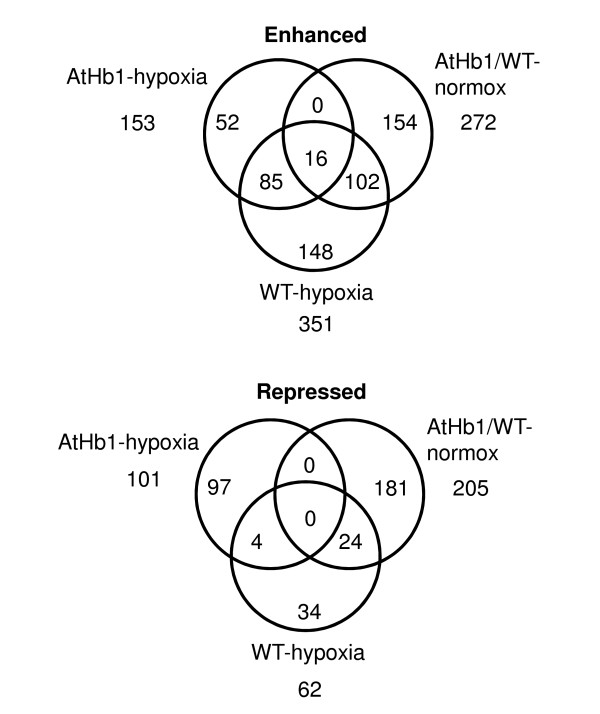
**Venn diagrams showing overlap of differentially expressed genes due to *AtHb1 *overexpression and genes involved in the hypoxic response of WT and/or *AtHb1 *plants**. Overlap of differentially expressed genes was identified using the Venn Super Selector of the web-based tool BAR (http://bbc.botany.utoronto.ca/).

Under hypoxic stress treatment, a significantly lower number of transcripts exhibited altered expression in *AtHb1 *compared to WT (254 and 413 genes, respectively). Consequently, the stress response observed in *AtHb1 *was much reduced, especially in regulatory/signaling pathways, but also for specific pathways in primary metabolism. Transcriptional alterations in WT upon hypoxia partly shared a commonality with those induced by *AtHb1 *overexpression under control conditions, or with transcripts additionally induced in *AtHb1*-overexpressing plants after hypoxia (Figure 4). The moderate hypoxic response in seeds of transgenic plants, in combination with genes induced by *AtHb1 *overexpression that have been shown to be implicated in the WT hypoxia response, points to a kind of "pre-adaptation" to oxidative stress. Among the differences between the two genotypes in their hypoxic responses, several biological processes stand out, namely, stress-related signaling, redox pathways and primary/energy metabolism (Figure 2, Additional file 4). These differences are discussed in detail below.

First, hypoxia induced stress-related signaling and redox pathways in WT. GO analysis for functional assignments of upregulated genes showed strong overrepresentation of responses to abiotic/biotic stress and other biological processes related to stress responses, especially responses to ABA and JA. Evaluation of promoter motifs within the 5'-flanking regions of hypoxia-induced genes revealed that W-box, ABRE, DREB, G-box, MYC2, MYCATERD1, GADOWNAT, Z-box, I-box and Evening Element motifs were significantly overrepresented. This finding is significant because almost all of these recognition sites have been implicated in hormone signaling (ABA, ethylene) and in general stress responses. In addition to these changes in hormone signaling pathways, transcripts directly involved in biosynthesis of ABA, ethylene, JA and SA were commonly upregulated in WT. In contrast genes related to SA, GA and ABA metabolism were not induced by hypoxia in *AtHb1 *plants. In fact, a strong repression of ABA synthesis/signaling was evident from the down regulation of *NCED4 *and several ABA-responsive genes, among them *ATEM6 *and *AtHVA22b *(which were already induced under control conditions by *AtHb1 *overexpression). In addition, ABRE binding site motifs were enriched in the set of downregulated genes in *AtHb1 *plants after hypoxia (Table 3). Another striking difference between the genotypes is the opposite regulation of transcripts encoding the gibberellin regulated proteins 2 and 3 (GASA 2, 3); they are highly upregulated in the WT after hypoxic treatment whereas a strong repression was observed in transgenic seeds. Calcium signaling seems to play a role in the hypoxic response of WT, as indicated by the upregulation of six transcripts encoding calmodulins and calmodulin binding proteins, accompanied by an induction of calcium dependent protein kinase and the plastidic *Ca^2+^-ATPase1 *(*ACA1*, At1g27770). The transcriptional activation of calmodulins which are the primary calcium receptors in plant cells and calcium binding proteins, could serve as substrate for phosphorylation by calcium dependent protein kinases, then activating transcription factors by phosphorylation. Altogether this points to existing calcium dependent signaling pathways in the hypoxia response in wild type seeds, which were not observed in *AtHb1 *overexpressors.

The second major difference between *AtHb1*-overexpressing plants and WT concerned primary and energy metabolism. Hypoxia induced multiple changes in transcripts related to these processes in WT, but only moderate changes in *AtHb1 *plants. For example, in WT we encountered a clear induction of glycolysis and fermentation (*FBP aldolase, PFK, PDC1, ADH*1) as well as strongly induced nitrogen assimilation as suggested by preferential expression of *NIA2 *and *NiR1*. In WT, cell wall metabolism was downregulated as evidenced by repression of six transcripts encoding pectinesterases and four encoding polygalacturonases, indicating that cell wall metabolism is one of the key processes affected by hypoxia. Induction of carbonic anhydrases and genes implicated in lipid degradation and the glyoxylate cycle (malate synthase, isocitrate lyase) was apparent in the WT response but not in *AtHb1 *plants. The activity of transporter genes is directly linked to primary metabolism. The strong induction of genes encoding proline transporter, POT as well as TIP1.2 and TIP3.2 is also restricted to the hypoxia response in WT and might reflect a higher demand for remobilizing storage compounds and thus, indicating nutrient depletion in WT. The alterations observed in the transgenic plants were restricted to upregulation of glycolysis/fermentation (*PFK, PDC1, ADH1*) and a few transcripts related to cell wall degradation.

### *AtHb1 *plants show less pronounced metabolic adjustment under transient hypoxia

**Figure 5:**
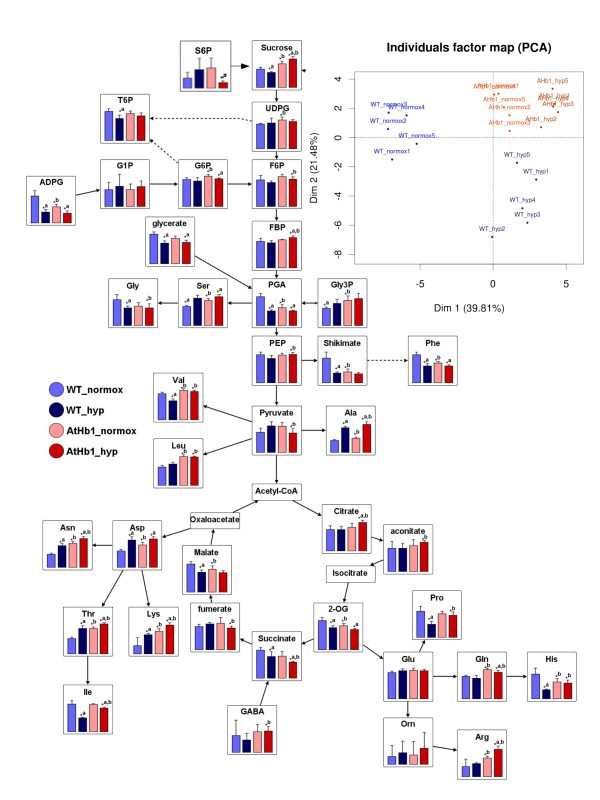
**Metabolite patterns in seeds of *AtHb1*-overexpressing and WT plants under control conditions (21 kPa O2) and moderate hypoxia (10.5 kPa O2) visualized by VANTED software **[75]. "*a" indicates statistically significant differences after hypoxic treatment in each genotype, "*b" indicates statistically significant differences between the genotypes under control and hypoxic conditions (t-test, p < 0.05). Mean values ± standard deviation are presented (data in Additional file 9). The insert shows results of a principal component analysis of the metabolite data set. 20 samples in two dimensional space are given, where the names are coloured according to the 4 different sample types (WT and *AtHb1*, under either control or hypoxic conditions; with 5 biological replicates each).

**Figure 6:**
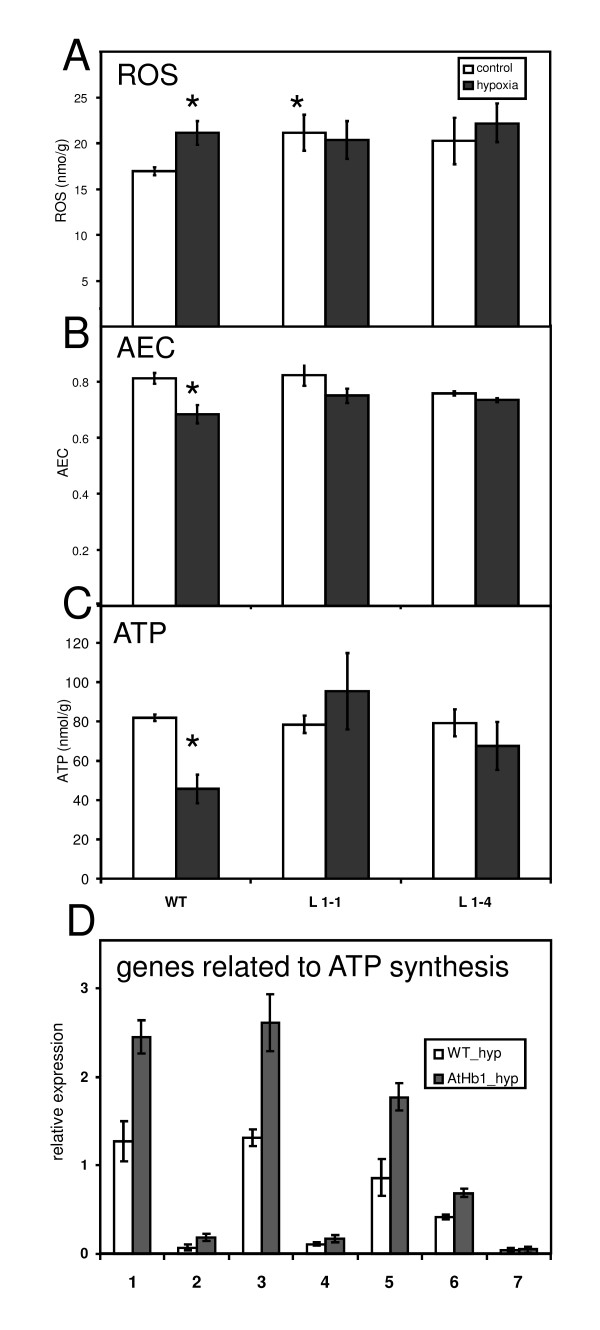
**Effects of *AtHb1 *overexpression on energy metabolism of seeds. Plants were grown under control conditions and moderate hypoxia**. (A) Levels of hydrogen peroxide (H2O2), (B) Adenylate energy charge (AEC) and (C) ATP levels. (D) Relative expression levels of genes involved in ATP synthesis quantified by qRT-PCR after hypoxic treatment. 1 - ATPase (At2g21870), 2 - ATPase (Chl) (At1g15700), 3 - NADH-DH (At5g47890), 4 - NADH:ubi (At5g18800), 5 - COX5C (At5g61310), 6 - COX5C (At3g62400), 7 - AOX1 (At1g32350). Mean values ± standard deviation are presented (n = 5); asterisks indicate statistical significant differences according to a student's t-test (p < 0.05, A-C).

The steady state level of amino acids, sugars, metabolic intermediates and H2O2 were measured in seeds/siliques of both genotypes under control and hypoxic conditions. Under control conditions, the levels of phosphoglycerate and ADP-glucose (starch precursor) were higher in WT versus *AtHb1 *plants, while sucrose and UDP-glucose (cell wall precursor), showed elevated levels in *AtHb1 *plants (Figure 5, values are given in Additional file 9). Remarkably, the levels of many metabolites changed after hypoxic treatment in WT but were barely altered in *AtHb1 *plants. In WT plants only, the levels of T6P and sucrose dropped significantly, while pyruvate increased (indicative of enhanced glycolytic flux and/or a partial block of the TCA cycle). Altogether, the metabolite profiles of the two genotypes illustrated a strong metabolic adjustment in WT in response to moderate hypoxia, whereas in *AtHb1 *only marginal changes were detected. This differential response was clearly visualized using principal component analysis (PCA; insert in Figure 5). Transcript data hinted at shifts in ROS metabolism in transgenic plants and in the hypoxic response of WT. Measurements of H2O2 levels in both genotypes under control and hypoxic conditions are consistent with transcriptional activities of H2O2 generating and scavenging enzymes. Higher concentrations in *AtHb1 *seeds/siliques compared to WT under control conditions (Figure 6A) correlate with preferential expression of *MnSOD1 *and glutathione-S-transferases. Upon hypoxia, H2O2 levels in WT increased but were unchanged in *AtHb1 *seeds. Activation of *respiratory burst oxidase homologue D, MnSOD1*, redoxins, three glutathionine-S-transferases and *alternative oxidase 1D *(*AOX1D*, At1g32350) in WT indicates an enhanced ROS metabolism under hypoxia.

### Overexpression of *AtHb1 *promotes respiration and maintains the energy status under transient hypoxia

To investigate changes in energy metabolism we measured the respiratory activity of developing seeds. Under control conditions respiration rates were similar in both genotypes (1.7 ± 0.2 pmol/µg embryo min). However, under hypoxia, respiration in *AtHb1 *plants (line 1-1, 1.05 ± 0.14 pmol/µg min) was about 40% higher than in WT (0.73 ± 0.13 pmol/µg min) pointing to a higher energy supply in the former. Indeed, both the adenylate energy status (AEC = (ATP+0.5ADP)/(ATP+ADP+AMP)) and total ATP levels were elevated in *AtHb1 *versus WT under hypoxia (Figure 6B-C).

Direct comparison of microarray data from the two genotypes under hypoxic conditions identified only the gamma-subunit of the chloroplast ATPase to be significantly upregulated in *AtHb1 *seeds. Screening our dataset for other differentially expressed transcripts involved in electron transport chain/ATP synthesis, we found five other transcripts, encoding ATP synthase, NADH dehydrogenase, NADH:ubiquinone oxidoreductase, cytochrome C oxidoreductase subunit 5c (COX 5C), with a tendency to higher expression in *AtHb1 *seeds under hypoxia (fold-changes between 1.4 and 1.64 and p-values < 0.05). These transcripts were found by qRT-PCR analysis to be nearly doubled in the *AtHb1-*overexpressing plants compared to WT (Figure 6D). Altogether, our data suggest that *AtHb1 *overexpression enables the seed to respire at higher rates especially under hypoxia, thereby increasing the ATP supply.

## Discussion

**Figure 7:**
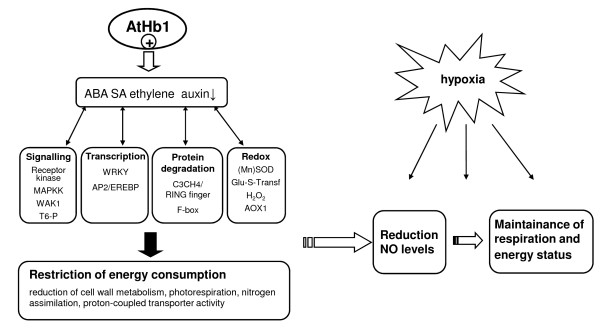
**Hypothetical model of pathways coordinated by *AtHb1 *overexpression and its effects on hypoxic stress responses**. Differences compared to WT as deduced from transcriptome, metabolite and physiological analyses are highlighted.

Although non-symbiotic Hbs have been widely used in plants to improve tolerance against different stresses, and overexpression of plant Hbs showed beneficial effects on energy status and growth under oxygen limitation [3,6,7], global information about the molecular mechanisms of *AtHb1 *function is missing. In this study, we present the first analysis of the underlying molecular mechanisms of *AtHb1 *function and signaling. The hypothetical model deduced from transcriptome, metabolite and physiological analyses summarizes the main effects of *AtHb1 *overexpression in seeds (Figure 7). Two different aspects should be considered when *AtHb1 *is overexpressed in seeds. First, under normal growth conditions the *AtHb1 *gene is barely expressed and thus, its overexpression itself might affect seed metabolism. Second, non-symbiotic hemoglobins, such as *AtHb1*, are able to degrade endogenously formed NO [7,8], which itself can act as a signal molecule. Thus, perception of the nitric oxide level in the seed might be altered due to the enzymatic scavenging of NO.

### *AtHb1 *overexpression induces stress-related signaling pathways and limits energy-consuming pathways

Under control conditions, *AtHb1 *overexpression activated several stress-related hormonal and signaling pathways. The fact that hormones and other components of signal transduction cascades work downstream of *AtHb1 *suggests that *AtHb1 *represents a high ranking signaling component with broad impact on regulatory networks. Most prominent was the induction of ABA synthesis/signaling, and the general repression of auxin transport/signaling. Evidence for induced ethylene and SA signaling came from induced MAPKK and WAK1-mediated signaling routes.

ROS formation also seems to be part of the *AtHb1 *signaling cascade as transcripts involved in formation and detoxification of H2O2 were clearly upregulated. Higher H2O2 levels in *AtHb1 *seeds confirmed the transcriptional activities. The pronounced upregulation of these stress-related signaling pathways might act in combination to "pre-adapt" the seeds to hypoxic stress. A role for plant non-symbiotic hemoglobins in redox regulation by improving the antioxidant status was previously hinted at by studies of alfalfa root cultures overexpressing a non-symbiotic hemoglobin [40]. *Hb1*-overexpressing lines revealed increased ascorbate levels as well as higher activity of enzymes involved in ROS removal. An enhanced oxidative stress tolerance during seed germination of *Arabidopsis *was induced by seed-specific overexpression of antioxidant genes [41]. Overexpression of MnSOD and/or combination with other genes encoding antioxidant enzymes during seed development and germination increased tocopherol contents and antioxidant capacities in mature seeds indicating beneficial effects of activated redox-related pathways on oxidative stress tolerance.

Alterations in transcriptional networks were accompanied by changes in primary metabolism. Cellulose synthesis, deposition of pectin fragments, incorporation of arabinose-derived sugars and glycosyl-transferring reactions all require energy and use activated nucleotide sugars. Thus, cell wall metabolism is clearly dependent on the energy and carbon status of cells. The decrease in transcripts related to cell wall metabolism in *AtHb1 *plants was the most prominent finding. The analysis of gene-to-gene interactions (MRNET approach) indicates that *AtHb1*-mediated downregulation of the hub gene *FLA13 *is of central importance for the proposed changes in cell wall metabolism. Its downregulation might eventually affect cell elongation, energy usage and carbon partitioning. Downregulation of cell wall metabolism might represent a major strategy to reduce energy (as well as carbon) consumption. Higher concentrations of UDP-glucose (precursor for cell wall synthesis) and sucrose support this idea.

Consistent with such energy saving adjustments is the transcriptional repression of proton-coupled transporters and photorespiration. Both require energy in the form of ATP, and thus, their repression implies a reduction in energy consumption. Another striking feature was the downregulation of *NIA2 *and *NiR1 *by *AtHb1 *overexpression under control conditions. While this might indicate lower nitrate assimilation (which imposes a high energy demand), the level of free amino acids was not reduced in transgenic seeds but rather elevated. The shift in *NIA2/NiR1 *expression could also be linked to NO signalling, because NIA can produce NO from nitrite [42-44]. High NO concentrations correlate with NIA activation and high nitrite levels [45,46]. Genetic studies using the *nia1nia2 *double mutant indicate that NIA is a major enzymatic source of NO formation in plants [47]. Subsequently, the coordinated downregulation of *NIA2/NiR1 *due to *AtHb1 *overexpression could prevent the accumulation of nitrite and subsequent NO formation. This would contribute to the lower steady state NO levels in the transgenics (beside the NO scavenging function of *AtHb1*).

Transcripts encoding key enzymes of photorespiration (GOX, SHMT4) were downregulated by *AtHb1 *overexpression. Photorespiration results in a net loss of fixed carbon and energy. The apparent repression of this pathway is a further indication for the energy-saving mode of metabolism. The preferential expression of the *β-carbonic anhydrase1 *in WT might also be related, as this enzyme is known to control CO2 availability to Rubisco and thereby regulate photorespiration [48,49].

Overall, alterations in the metabolism of *AtHb1*-overexpressing seeds point to an energy-saving mode of metabolism.

### NO formation and signaling pathways are repressed by *AtHb1 *overexpression resulting in improved respiration under stress

*AtHb1*-overexpressing seeds showed a much attenuated hypoxic response, with only some of the characteristic pathways being induced under hypoxia (e.g. enhancement of ethylene signaling, JA metabolism, redox-related transcripts and MYB transcription factors). Of particular note is the repression of the ABA response in the *AtHb1 *overexpressors, which contrasts with the strong induction observed in WT plants. Major differences were also obvious in calcium-dependent and GA-mediated signaling pathways. Both seem to play a much less significant role when compared to WT (e.g. *GASA2/GASA3 *showed the opposite responses in the two genotypes). Similarly, at the metabolite level, only minor alterations were apparent in response to hypoxia (in contrast to WT).

Another major difference in the hypoxic response of the two genotypes was the reduction of NO levels in *AtHb1*-overexpressing seeds. This agrees with previous findings [4,50] and could be attributable to *AtHb1*-mediated degradation of NO [7] and/or the restriction of NO formation via transcriptional downregulation of NIA2/NiR1. As *AtHb1 *overexpression represses *NIA2 *and *NiR1 *activity under control conditions and especially after hypoxia treatment it could be concluded that NO formation is strictly prevented by the reduction of NO precursors (e.g. nitrite). Studies from Wang et al. [51] provided evidence that NIA2 is responsible for stress-induced NO formation in *Arabidopsis *roots. They demonstrated that NIA2 is phosphorylated by MAPK6 leading to an increase of NR activity and subsequently NO formation. MAPK3 also interacted with NIA1 and 2 in the yeast two-hybrid system implying a role for activation of NIA activity. The transcriptional upregulation of MAPK3 and NIA2 in WT seeds after hypoxia is in agreement with this finding. Possibly MAPK3 represents a seed-specific transducer of environmental stimuli whereas MAPK6 is predominantly involved in NO biosynthesis in roots. Assuming that overexpression of *AtHb1 *lowered levels of NO in planta, the present approach enabled us to discriminate between the more general hypoxia response and the target genes specifically induced by higher NO levels in WT. The direct comparison of the transcriptome of both genotypes under hypoxic conditions (Figure 2, column 2) revealed differences which might be specifically attributed to NO signaling. Calcium signaling is linked to NO signaling pathways [52,53] and possibly directly involved in the regulation of hemoglobin expression [54]. NO induces a rapid increase in calcium concentrations [55,56], and vice versa [53]. This relationship was found in transgenic plants, where both NO levels and calcium-dependent signaling were lowered compared to WT. Hints for a crosstalk of NO and GA signaling came from studies with isolated aleurone cells of Arabidopsis. Bethke et al. [57] showed that NO works upstream of GA in a signaling pathway, supporting our results that GA is possibly linked to higher NO levels in WT. NO-responsive genes in Arabidopsis were identified by microarray analyses using the synthetic NO donors SNP and NOR-3 [58,59]. Among them genes involved in calcium signaling (calmodulins, calcium binding proteins), sugar and peptide transporters as well as glycosyltransferases which are preferentially expressed in the WT under hypoxic conditions. Based on our genetic approach we can separate these transcripts from transcripts of stress-related pathways (which are part of the hypoxia response without NO synthesis/accumulation).

According to our working hypothesis, lower NO levels in *AtHb1*-overexpressing seeds were expected to stimulate respiration because NO inhibits cytochrome C oxidase [60,61]. In fact, seeds of the transgenic plants retained respiratory activity as well as higher expression of *COX 5C *transcripts under hypoxia, whereas the WT switched to a "stress" mode. Congruently, there was a preferential expression of other genes related to electron transport chain/ATP synthesis in *AtHb1 *plants. Combined with repression of energy-demanding processes (e.g. cell wall metabolism) this eventually leads to an improved energy status of cells in *AtHb1-*overexpressing seeds.

## Conclusions

According to our previous hypothesis [5,10], NO integrates energy and carbon metabolism, enables the seed to balance its oxygen demand and to avoid self-anoxia. *AtHB1 *overexpression and/or the subsequent decline in endogenous NO levels set the seed in a state of 'alarm'. This is characterized by changes in hormone metabolism, induction of specific signaling pathways and transcription factors, targeted protein degradation and changes in redox-related pathways. These alterations resulted in repression of energy-demanding processes, particular in cell wall metabolism, reflecting the pre-adaptation to (hypoxic) stress. Thus, the protective role of *AtHb1 *overexpression can be regarded as a positive stress (tential 'eustress'). This became even more evident upon stress treatment where seeds of transgenics showed an attenuated stress response. *AtHb1 *overexpression enabled the seed to respire at higher rates, which was likely related to the reduction of endogenous NO levels, and helped to maintain the energy status of cells under stress. These properties might be beneficial for daily life, because seed development is prone to regular oxygen deficiency and the day/night transition causes strong fluctuations in the seeds' oxygen status [12]. Such transient stress conditions occur daily and necessitate the adjustment of respiratory activity and metabolism. Subsequently, pre-adapted transgenic seeds might have advantages under "normal" growth conditions, driving metabolism more energy-efficient, and eventually accumulating higher seed biomass.

## Methods

### Generation of transgenic plants, growth conditions and treatment

The coding region of *AtHb1 *(At2g16060) was PCR-amplified (F-GGATCCGAGGTTGTGAAATATTATGGAG and R-GGATCCTAGGATTTTGGAATGCACACTA BamHI sites underlined) using a full-length *AtHb1 *clone (kindly provided by P. Geigenberger, LMU Munich, Germany). After subcloning into the pCR4-TOPO vector, *AtHb1 *was introduced into the modified binary vector pBAR between the LeB4 promoter [19] and OCS terminator. After sequencing, the construct was mobilized in *Agrobacterium tumefaciens *EHA105 and used for transformation of *Arabidopsis thaliana *Col-0 plants by floral dipping [62]. Homozygous plants were selected on phytagar plates with ½ Murashige and Skoog medium [63] supplemented with phosphinothrycin (50 µg ml-1) and characterized by Southern blot analysis. Plants were grown at 22°C under a 16/8-h photoperiod, with a relative air humidity of 60% and an approximate light intensity of 100-150 µmol photons m^-2 ^second^-1^.

Hypoxic and normoxic treatments were carried out with transgenic (T3) and WT plants 45 days after germination (DAG) corresponding to the mid phase of maturation ~11/12 days after pollination. Plants were aerated with a gas mixture containing 10.5% O2 (composed of a 1:1 mixture of ambient gas and N2) or ambient gas containing 21% O2 for control samples in darkness. After one hour, plants were decapitated and immediately frozen in liquid N2. About 70-80 siliques of the same developmental stage were dissected in liquid nitrogen and pooled for one biological replicate. Both hypoxic and control treatment runs were repeated twice to provide biologically replicated samples. From the pool of biological replicates sample material was used for microarray and metabolite analyses.

### Northern blot and RT-PCR analysis

Isolation of total RNA from siliques/seeds was performed according to Heim et al. [64]. For northern blot analysis, 10 µg total RNA were blotted on nylon membrane (Hybond-N+, Amersham) and hybridized with a [^32^P]-labelled 635-bp fragment of *Arabidopsis AtHb1 *cDNA. A 25S rDNA fragment was used as loading control.

For cDNA synthesis, isolated total RNA was treated with RNAse free TURBO DNase (Ambion) and 1 µg RNA was reverse transcribed using oligo(dT) primer and SuperScript III reverse tanscriptase (Invitrogen, Karlsruhe, Germany). Gene-specific primers for *AtHb1 *were used in the PCR reactions.

### RNA preparation and microarray hybridization

Total RNA was isolated from intact siliques using a GENTRA kit (Biozym, Germany) according to the manufacturer's instructions. RNA was further purified using an RNeasy Kit (Qiagen) and subjected to DNAse digestion (Qiagen). Total RNA was quantified using a NanoDrop ND-1000 UV-Vis spectrophotometer (Nanodrop Technology) and RNA quality was assessed using an Agilent 2100 Bioanalyzer (Agilent Technology). Three independent biological replicates of each genotype (WT, *AtHb1*) and treatment (hypoxia, control) were hybridized to Affymetrix ATH1 *Arabidopsis *GeneChips (n = 12). Preparation of labelled cRNA and hybridization of oligonucleotide chips was performed at the Deutsches Ressourcenzentrum für Genomforschung (Germany).

### Data analysis

Data were processed with the Affymetrix MicroArray Suite software package (MAS 5.0) and the resulting CEL files were analyzed using Bioconductor packages (http://www.bioconductor.org/) in R (http://cran.at.r-project.org/). Data were normalized using the Robust Multi-array Average (RMA) method [65]. Analysis of differentially expressed genes in the different comparisons was performed with the *LIMMA *package using the RMA normalized expression values [66]. The Benjamini and Hochberg method was selected to adjust p-values for multiple testing and to determine false discovery rates (FDRs) [67]. Genes were deemed to be differentially expressed only when (1) calculated p-value was < 0.05, (2) mean of the signal log2 ratio was > 1, and (3) signal intensities of probe sets from at least two of the three biological replicates were designated as "present" calls in the PMA analysis. Genes differentially expressed in all of the comparisons (i.e. in at least one of the four comparisons) were used as data sets for the subsequent clustering and gene category analyses.

K-means clustering was performed by means of the TMeV software package using log2 signal ratio data. The MapMan visualization tool was used for functional characterization of differentially expressed genes. Enrichment analysis of Gene Ontology (GO) terms for differentially expressed genes was performed as in Horan et al. [68]. For identification of conserved motifs in the promoters of differentially expressed genes the online tool Athena (http://www.bioinformatics2.wsu.edu/cgi-bin/Athena/cgi/analysis_select.pl) was used with the default settings.

All microarray data from this study have been deposited in NCBI Gene Expression Omnibus (accession number GSE23846).

### Reconstruction of the gene regulatory network

Inferring regulatory networks from microarray data was done based on the information theoretic approach MRNET (package *minet *Bioconductor/R) using the top 20 of differentially expressed genes (given in Additional file 10). MRNET is based on the maximum relevance/minimum redundancy algorithm. The algorithm starts with computing the pairwise mutual information (MI) between all gene pairs. The resulting MI matrix is then manipulated to identify regulatory relationships and to reduce the number of false positives.

### Quantitative Real-Time PCR

RNA preparations from microarray experiments were used for cDNA synthesis (see above). The Power SYBR Green PCR mastermix was used to perform reactions in an ABI 7900 HT Real-Time PCR system (Applied Biosystems, CA, USA). Data were analyzed using SDS 2.2.1 software (Applied Biosystems). Five replicate measurements were conducted for each gene. Expression values were normalized with transcript levels of the *actin 2 *gene (At3g18780) and calculated as an arithmetic mean of the replicates. Dissociation curves confirmed the presence of a single amplicon in each PCR reaction. Log2 fold-changes were calculated after Livak and Schmittgen [69]. Efficiencies of PCR reactions were determined using LinRegPCR software (http://www.gene-quantification.de/download.html). A list containing primers for the tested genes is given in Additional file 11.

### Fluorescence detection assay for nitric oxide in embryos

Analysis of NO levels was done using DAF-2DA fluorescence detection [70]. Freshly isolated *Arabidopsis *embryos were incubated in 1 ml buffer solution containing: 50 mM sucrose, 10 mM KCL, 0.1 mM CaCl2, 10 mM MES-Tris (pH 5.6) and 50 µM DAF-2DA (Calbiochem, Germany). The buffer was aerated with 15 µM oxygen. After 1 h incubation, embryos were rinsed with fresh buffer to remove excess fluorophore. Fluorescence was analyzed using a laser scanning confocal microscope (510 Meta, Carl Zeiss, Jena, Germany).

### Respiratory oxygen uptake

About 100 *Arabidopsis *seeds were incubated in 2 ml buffer (100 mM sucrose, ¼ MS-medium, 10 mM MES-NaOH, pH 6.35). Gas tight closed vessels equipped with an oxygen sensor SP-PSt3 and connected to a Fibox 3 oxygen meter (PreSens Sensing GmbH, Regensburg, Germany) were used. Oxygen concentration in the samples was registered during a time period of 3 min. From recorded data the respiration rate of seeds was calculated by linear regression.

### Determination of metabolic intermediates, storage products and seed weight

Sugar-phosphates, nucleotide sugars and organic acids were extracted in chloroform/methanol (3:7 v/v) and measured by anion-exchange chromatography linked to tandem mass spectrometry [71]. For amino acid measurements 10 mg of powdered, frozen material was extracted in ethanol (80%, v/v), supplemented with 25 nmol norvaline as internal standard. Collected supernatants were vacuum-dried and resuspended in 250 µl water. Derivatization and separation of amino acids was performed according to Thiel *et al. *[72]. H2O2 was quantified using the Amplex Red Hydrogen Peroxide/Peroxidase Assay Kit (A22188; Molecular Probes, Invitrogen GmbH, Darmstadt, Germany) according to the manufacturer's instructions. Adenine nucleotides were measured as in Rolletschek *et al. *[73].

Average weight and number of mature seeds was determined in 4 independent batches of plants. In each batch, we used 5 individual plants per genotype, and counted the number of siliques per plant and the number of seeds per siliques (n = 10). From this we counted the total number of seeds per plant. Average seed weight was analysed in three generations (T3-T5) using an electronic microbalance (M2P, Sartorius, Göttingen, Germany). Total lipid of mature seeds was analyzed as fatty acid methyl esters by gas chromatography [74]. Total nitrogen and total carbon content were measured by elemental analysis (Vario EL3, Elementaranalysesysteme, Hanau, Germany).

## Authors' contributions

JT, HR and LB designed research. JT, HR, THN, RF, HT, MM and LB carried out research. JT and SF analyzed the data. JT, HR, LB and JEL wrote the paper. All author's have read and approved the manuscript.

## Supplementary Material

Additional file 1 **Validation of microarray data**. (A) Cluster dendrogram of normalized expression values (WT-wild type, HB-*AtHb1 *overexpression, H-hypoxic treatment, C-control, numbers indicate biological replicates). (B) Correlation of qRT-PCR and microarray data. Changes in gene expression of a selected set of 20 genes represented as log2 (hypoxia/control) derived from qRT-PCR and microarray hybridizations were compared. Correlation of gene expression data was measured in both genotypes. Accordingly, each gene is represented by two pairs of values. Click here for file

Additional file 2 **Clustering of differentially expressed genes**. K-means clustering of differentially expressed genes in all of the comparisons (see also Additional file 3) according to expression profiles (n = 8). Arrangement of comparisons into vertical columns is the same as described in the legend of Figure 2. Columns indicate the number of genes (no. Genes) per cluster, colours indicate increased (yellow) or decreased (blue) expression. Clusters 1-3 showed similar expression profiles of genes preferentially induced or repressed in transgenics compared to WT under control conditions (*AtHb1*/WT_normox) and genes implicated in hypoxic response in WT (WT_hyp/normox). Clusters 4-5 contained genes upregulated in both genotypes upon hypoxia (WT_hyp/normox and *AtHb1*_hyp/normox). In cluster 6, genes exclusively upregulated in WT after hypoxic treatment were monitored. Genes in clusters 7-8 were found to be upregulated in *AtHb1 *after hypoxia, but not in WT. Click here for file

Additional file 3 **List of differentially expressed genes**. List of differentially expressed genes in all of the comparisons. A total of 1,010 genes was identified as differentially expressed (log2 fold-change >1, p-val < 0.05). Click here for file

Additional file 4 **Differentially expressed genes organized by pathways**. Classification of functional groups was done using MapMan software. Annotation was confirmed using the TAIR locus history retrieval tool http://www.arabidopsis.org/tools/bulk/locushistory/index.jsp. Click here for file

Additional file 5 **Overrepresented GO terms of differentially expressed genes in each comparison**. Selected GOs were defined as enriched by p-values < e-06. Ontology, MF-molecular function, BP-biological process, CC-cellular compartment; n.e.-not enriched. Click here for file

Additional file 6 **Heat map display of differentially regulated genes of the ubiquitin proteasome**. Arrangement of comparisons into vertical columns is the same as described in the legend of Figure 2. Click here for file

Additional file 7 **Effects of *AtHb1 *overexpression on transcripts involved in primary metabolism under control and hypoxic conditions displayed by MapMan tool**. (A) *AtHb1 *vs WT under control conditions. (B) *AtHb1 *vs WT under hypoxia. Log2 ratios of genes are displayed using the colour code indicated. Blue, upregulation in *AtHb1*; red, upregulation in WT. Click here for file

Additional file 8 **Reconstructed network of gene-to-gene interactions for WT and transgenic plants**. Network analysis is based on the top 20 differentially expressed genes between the genotypes under control conditions. Colours of the nodes indicate upregulated (green) or downregulated (red) genes in *AtHb1 *versus WT. The colour of the lines indicates the degree of information flow between genes. Red indicates strong relationships between genes (gene information in Additional file 10). Click here for file

Additional file 9 **Metabolite levels of WT and *AtHb1*-overexpressing seeds under control and hypoxic conditions**. LC/MS measurements have been conducted with 5 biological replicates each (+/- SD). Click here for file

Additional file 10 **Top 20 of differentially expressed genes between WT and *AtHb1*-overexpressing plants under control conditions used for network analysis**. Click here for file

Additional file 11 **Oligonucleotide primers used for quantitative Real-Time PCR**. Click here for file
