# Supplementary figures and images for: Seed-specific elevation of non-symbiotic hemoglobin AtHb1: beneficial effects and underlying molecular networks in Arabidopsis thaliana
Source: BMC Plant Biol. 2011 Mar 15;11:48. doi: 10.1186/1471-2229-11-48 (PMC3068945; doi:10.1186/1471-2229-11-48)

## Slide 1
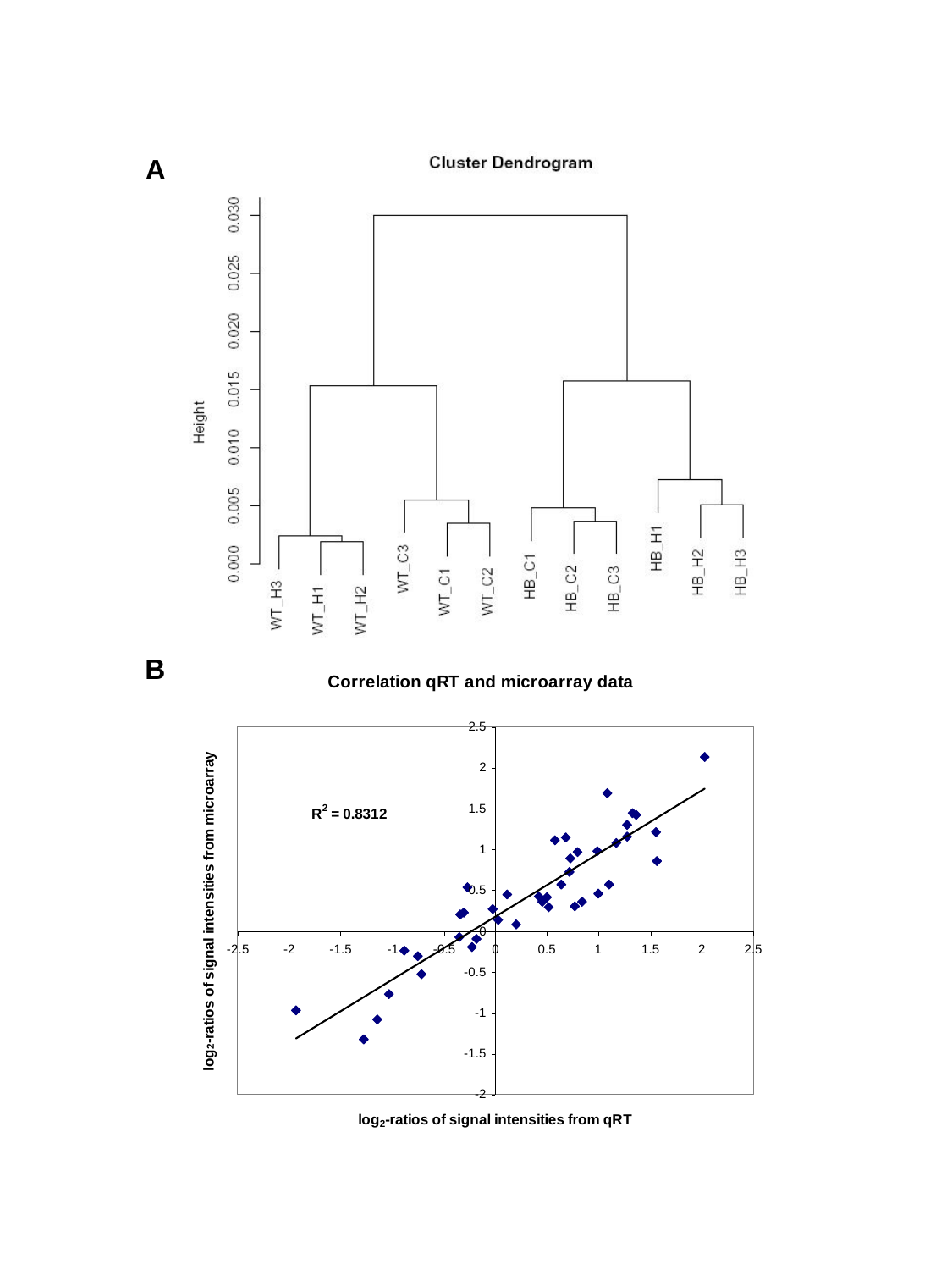

A
B

Supplement: Additional file 1 — Validation of microarray data. (A) Cluster dendrogram of normalized expression values (WT-wild type, HB-AtHb1 overexpression, H-hypoxic treatment, C-control, numbers indicate biological replicates). (B) Correlation of qRT-PCR and microarray data. Changes in gene expression of a selected set of 20 genes represented as log2 (hypoxia/control) derived from qRT-PCR and microarray hybridizations were compared. Correlation of gene expression data was measured in both genotypes. Accordingly, each gene is represented by two pairs of values. [file 1471-2229-11-48-S1.PPT]
