# Supplementary material for: Seed-specific elevation of non-symbiotic hemoglobin AtHb1: beneficial effects and underlying molecular networks in Arabidopsis thaliana
Source: BMC Plant Biol. 2011 Mar 15;11:48. doi: 10.1186/1471-2229-11-48 (PMC3068945; doi:10.1186/1471-2229-11-48)
Supplement: Additional file 2 — Clustering of differentially expressed genes. K-means clustering of differentially expressed genes in all of the comparisons (see also Additional file 3) according to expression profiles (n = 8). Arrangement of comparisons into vertical columns is the same as described in the legend of Figure 2. Columns indicate the number of genes (no. Genes) per cluster, colours indicate increased (yellow) or decreased (blue) expression. Clusters 1-3 showed similar expression profiles of genes preferentially induced or repressed in transgenics compared to WT under control conditions (AtHb1/WT_normox) and genes implicated in hypoxic response in WT (WT_hyp/normox). Clusters 4-5 contained genes upregulated in both genotypes upon hypoxia (WT_hyp/normox and AtHb1_hyp/normox). In cluster 6, genes exclusively upregulated in WT after hypoxic treatment were monitored. Genes in clusters 7-8 were found to be upregulated in AtHb1 after hypoxia, but not in WT. [file 1471-2229-11-48-S2.PPT]

## Slide 1
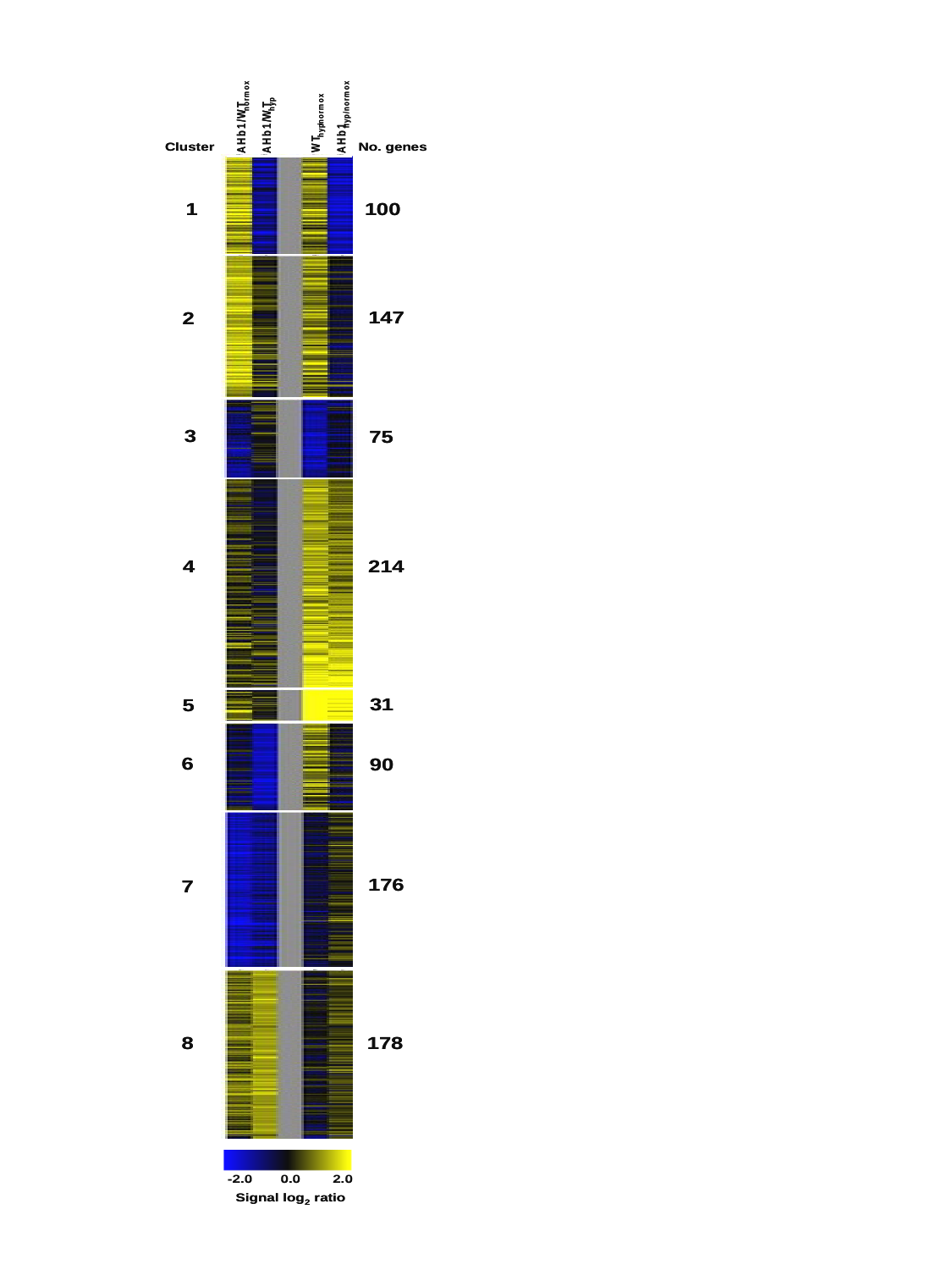

Figure 2. K-means clustering of differentially expressed genes in all of the comparisons according to expression profiles. Each comparison is arranged into vertical columns in the following order: column 1, AtHb1 overexpression versus wild type under control conditions; column 2, comparison of both genotypes under hypoxic conditions; column 3, wild type under hypoxia versus wild type under normoxia; column 4, transgenic under hypoxia versus transgenic under normoxia. Columns 2 and 3 are separated by grey column. A total of 1011 genes was extracted from the data base by applying a fold-change of >2 (log2 >1) and a p-value of <0.05 in all of the comparisons. Columns indicate the number of genes (no. Genes) per cluster, colours indicate inreased (yellow), decreased (blue) or unchanged expression (black).
