# Supplementary material for: Seed-specific elevation of non-symbiotic hemoglobin AtHb1: beneficial effects and underlying molecular networks in Arabidopsis thaliana
Source: BMC Plant Biol. 2011 Mar 15;11:48. doi: 10.1186/1471-2229-11-48 (PMC3068945; doi:10.1186/1471-2229-11-48)
Supplement: Additional file 5 — Overrepresented GO terms of differentially expressed genes in each comparison. Selected GOs were defined as enriched by p-values < e-06. Ontology, MF-molecular function, BP-biological process, CC-cellular compartment; n.e.-not enriched. [file 1471-2229-11-48-S5.DOC]

| **Additional file 5. Overrepresented GO terms of differentially expressed genes in each comparison.**  Selected GOs were defined as enriched by p-values <e-06. Ontology, MF-molecular function, BP-biological process, CC-cellular compartment; n.e.-not enriched. | | | | |
| --- | --- | --- | --- | --- |
| GO | ontology | p-Value of enrichment | | |
|  |  | **control** | **hypoxia** | |
|  |  | *AtHb1* vs WT upregulated | | *AtHb1* vs WT upregulated |
| response to stimulus | BP | 6,46e-12 | | no enrichment in GO terms |
| response to water | BP | 1,45e-11 | |
| response to chemical stimulus | BP | 5,86e-10 | |  |
| response to stress | BP | 1,29e-08 | |  |
| response to water deprivation | BP | 1,43e-08 | |  |
| response to abscisic acid stimulus | BP | 2,58e-08 | |  |
| response to endogenous stimulus | BP | 1,89e-07 | |  |
| response to abiotic stimulus | BP | 1,94e-07 | |  |
| multicellular organismal process | BP | 1,99e-07 | |  |
| embryonic development | BP | 7,47e-07 | |  |
|  |  | *AtHb1* vs WT downregulated | | *AtHb1* vs WT downregulated |
| water transporter activity | MF | n.e. | | 6,21e-09 |
| water channel activity | MF | n.e. | | 6,21e-09 |
| oxidoreductase activity | MF | n.e. | | 5,72e-07 |
| unidimensional cell growth | BP | 4,29e-10 | | 8,46e-07 |
| cellulose and pectin-containing cell wall organization and biogenesis | BP | 4,75e-10 | | 4,39e-09 |
| cell growth | BP | 1,12e-09 | | n.e. |
| cell wall organization and biogenesis | BP | 1,59e-09 | | 5,95e-07 |
| regulation of cell size | BP | 1,64e-09 | | n.e. |
| external encapsulating structure organization and biogenesis | BP | 1,78e-09 | | n.e. |
| cellulose and pectin-containing cell wall loosening | BP | 5,11e-09 | | 3,42e-09 |
| syncytium formation | BP | 5,28e-09 | | 4,78e-07 |
| growth | BP | 7,64e-09 | | n.e. |
| cellulose and pectin-containing cell wall modification | BP | 9,59e-09 | | 6,44e-09 |
| response to auxin stimulus | BP | 4,27e-07 | | n.e. |
| cell wall modification | BP | 9,73e-07 | | 6,30e-07 |
| multidimensional cell growth | BP | n.e. | | 6,23e-07 |
| cell wall modification during multidimensional cell growth | BP | n.e. | | 2,88e-08 |
| response to stimulus | BP | n.e. | | 4,96e-08 |
| cell wall | CC | 3,82e-15 | | 5,49e-11 |
| external encapsulating structure | CC | 5,25e-15 | | 7,03e-11 |
| membrane | CC | 4,25e-12 | | n.e. |
| cellulose and pectin-containing cell wall | CC | 1,12e-11 | | n.e. |
| anchored to membrane | CC | 4,74e-08 | | n.e. |
| cell | CC | 1,97e-07 | | n.e. |
| cell part | CC | 1,97e-07 | | n.e. |
| endomembrane system | CC | 8,20e-07 | | n.e. |
| membrane | CC | n.e. | | 2,25e-08 |
| extracellular region | CC | n.e. | | 9,25e-07 |

| name of GO | ontology | p-value of enrichment | |
| --- | --- | --- | --- |
|  |  | **hypoxia** | |
|  |  | WT upregulated | *AtHb1* upregulated |
| response to stimulus | BP | 1,25e-20 | 1,77e-11 |
| response to water | BP | 8,93e-16 | n.e. |
| defense response | BP | 1,23e-15 | n.e. |
| response to stress | BP | 1,11e-14 | 2,10e-09 |
| response to water deprivation | BP | 6,01e-14 | n.e. |
| response to chemical stimulus | BP | 7,57e-13 | 8,01e-10 |
| response to abiotic stimulus | BP | 8,13e-13 | n.e. |
| immune response | BP | 3,55e-12 | n.e. |
| immune system process | BP | 4,00e-12 | n.e. |
| response to biotic stimulus | BP | 5,14e-12 | n.e. |
| response to other organism | BP | 1,11e-10 | n.e. |
| regulation of defense response | BP | 1,96e-10 | n.e. |
| response to wounding | BP | 1,24e-09 | n.e. |
| multi-organism process | BP | 1,66e-09 | n.e. |
| response to external stimulus | BP | 1,7e-09 | n.e. |
| innate immune response | BP | 9,58e-09 | n.e. |
| regulation of response to stimulus | BP | 9,81e-09 | n.e. |
| response to temperature stimulus | BP | 1,12e-08 | n.e. |
| response to cold | BP | 2,22e-08 | n.e. |
| regulation of immune system process | BP | 4,11e-08 | n.e. |
| response to abscisic acid stimulus | BP | 8,87e-08 | n.e. |
| response to endogenous stimulus | BP | 9,37e-08 | 4,73e-07 |
| response to osmotic stress | BP | 2,21e-07 | n.e. |
| jasmonic acid mediated signaling pathway | BP | 6,54e-07 | n.e. |
| cell wall | CC | 5,08e-07 | n.e. |
| external encapsulating structure | CC | 6,26e-07 | n.e. |
| response to hormone stimulus | BP | n.e. | 8,34e-08 |
|  |  | WT downregulated | *AtHb1* downregulated |
| pectinesterase inhibitor activity | MF | 7,71e-09 | no enrichment in GO terms |
| enzyme inhibitor activity | MF | 1,89e-08 |
