# Supplementary material for: Seed-specific elevation of non-symbiotic hemoglobin AtHb1: beneficial effects and underlying molecular networks in Arabidopsis thaliana
Source: BMC Plant Biol. 2011 Mar 15;11:48. doi: 10.1186/1471-2229-11-48 (PMC3068945; doi:10.1186/1471-2229-11-48)
Supplement: Additional file 6 — Heat map display of differentially regulated genes of the ubiquitin proteasome. Arrangement of comparisons into vertical columns is the same as described in the legend of Figure 2. [file 1471-2229-11-48-S6.PPT]

## Slide 1
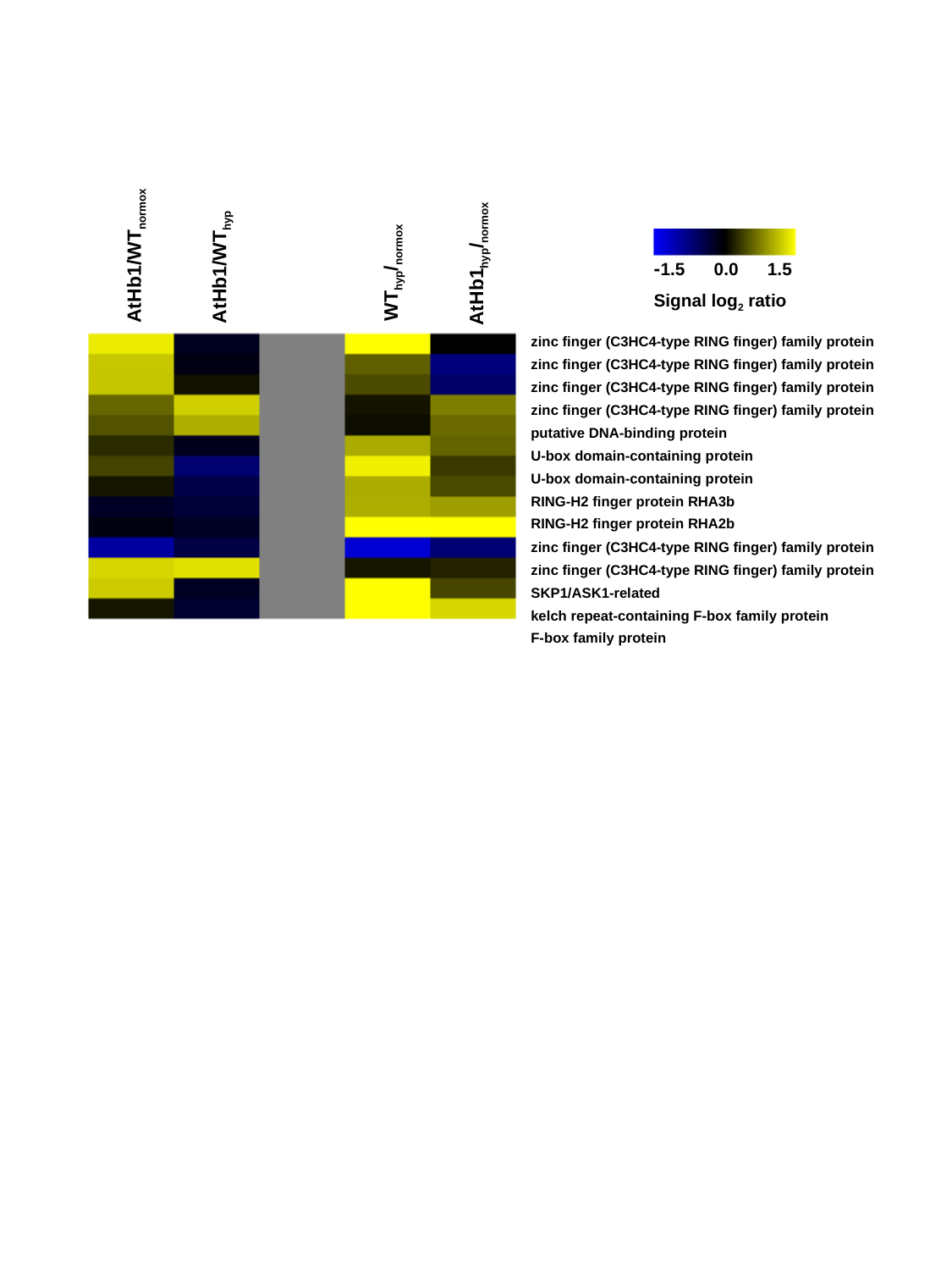

AtHb1hyp/normox
AtHb1/WThyp
AtHb1/WTnormox
WThyp/normox
-1.5 0.0 1.5
Signal log2 ratio
| zinc finger (C3HC4-type RING finger) family protein |
| --- |
| zinc finger (C3HC4-type RING finger) family protein |
| zinc finger (C3HC4-type RING finger) family protein |
| zinc finger (C3HC4-type RING finger) family protein |
| putative DNA-binding protein |
| U-box domain-containing protein |
| U-box domain-containing protein |
| RING-H2 finger protein RHA3b |
| RING-H2 finger protein RHA2b |
| zinc finger (C3HC4-type RING finger) family protein |
| zinc finger (C3HC4-type RING finger) family protein |
| SKP1/ASK1-related |
| kelch repeat-containing F-box family protein |
| F-box family protein |
