# Supplementary material for: Seed-specific elevation of non-symbiotic hemoglobin AtHb1: beneficial effects and underlying molecular networks in Arabidopsis thaliana
Source: BMC Plant Biol. 2011 Mar 15;11:48. doi: 10.1186/1471-2229-11-48 (PMC3068945; doi:10.1186/1471-2229-11-48)
Supplement: Additional file 7 — Effects of AtHb1 overexpression on transcripts involved in primary metabolism under control and hypoxic conditions displayed by MapMan tool. (A) AtHb1 vs WT under control conditions. (B) AtHb1 vs WT under hypoxia. Log2 ratios of genes are displayed using the colour code indicated. Blue, upregulation in AtHb1; red, upregulation in WT. [file 1471-2229-11-48-S7.PPT]

## Slide 1
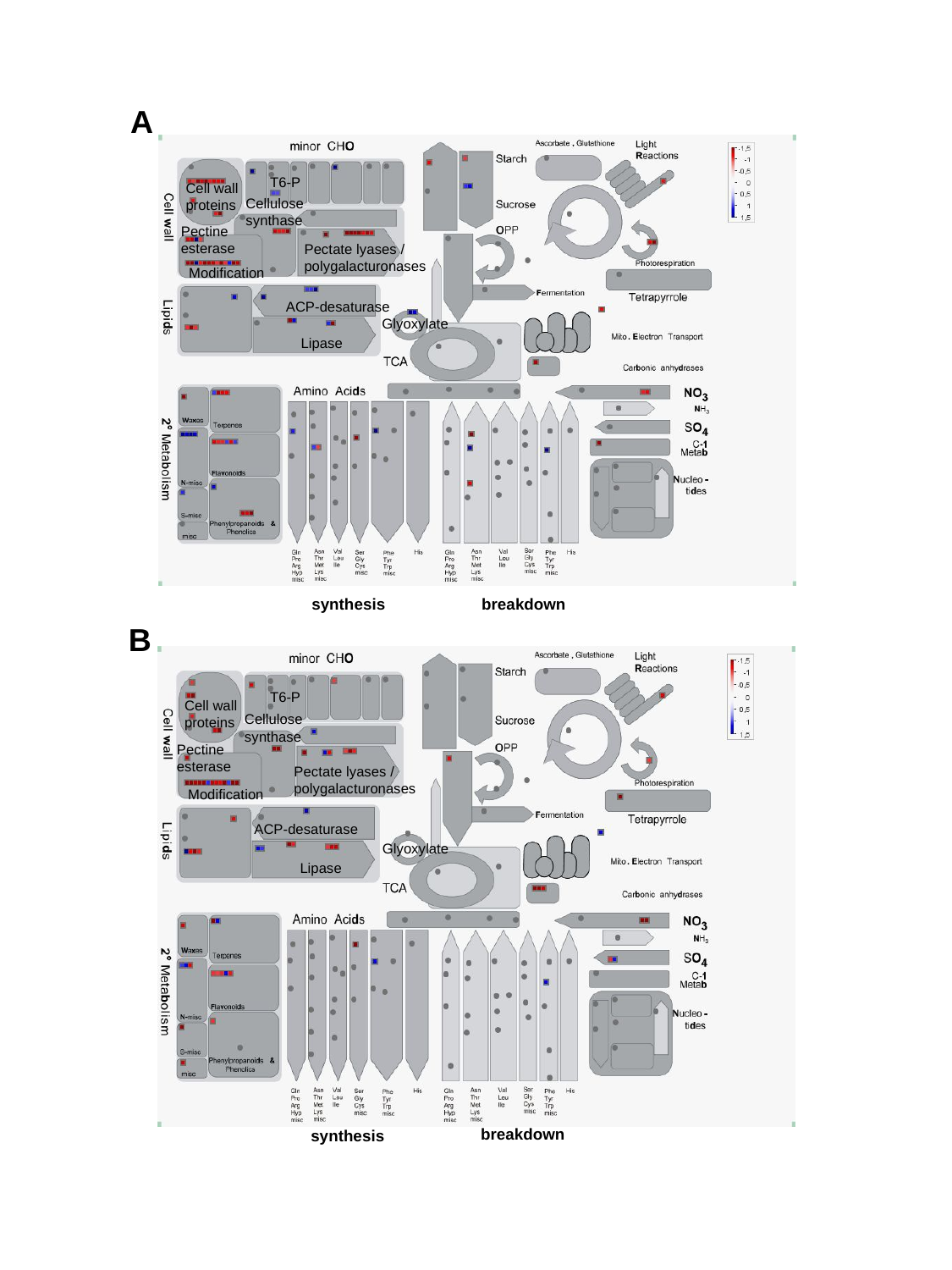

A
T6-P
Cell wall proteins
Cellulose
synthase
Pectine esterase
Pectate lyases / polygalacturonases
Modification
ACP-desaturase
Glyoxylate
Lipase
synthesis
breakdown
B
T6-P
Cell wall proteins
Cellulose
synthase
Pectine esterase
Pectate lyases / polygalacturonases
Modification
ACP-desaturase
Glyoxylate
Lipase
breakdown
synthesis
