# Supplementary material for: Seed-specific elevation of non-symbiotic hemoglobin AtHb1: beneficial effects and underlying molecular networks in Arabidopsis thaliana
Source: BMC Plant Biol. 2011 Mar 15;11:48. doi: 10.1186/1471-2229-11-48 (PMC3068945; doi:10.1186/1471-2229-11-48)
Supplement: Additional file 8 — Reconstructed network of gene-to-gene interactions for WT and transgenic plants. Network analysis is based on the top 20 differentially expressed genes between the genotypes under control conditions. Colours of the nodes indicate upregulated (green) or downregulated (red) genes in AtHb1 versus WT. The colour of the lines indicates the degree of information flow between genes. Red indicates strong relationships between genes (gene information in Additional file 10). [file 1471-2229-11-48-S8.PPT]

## Slide 1
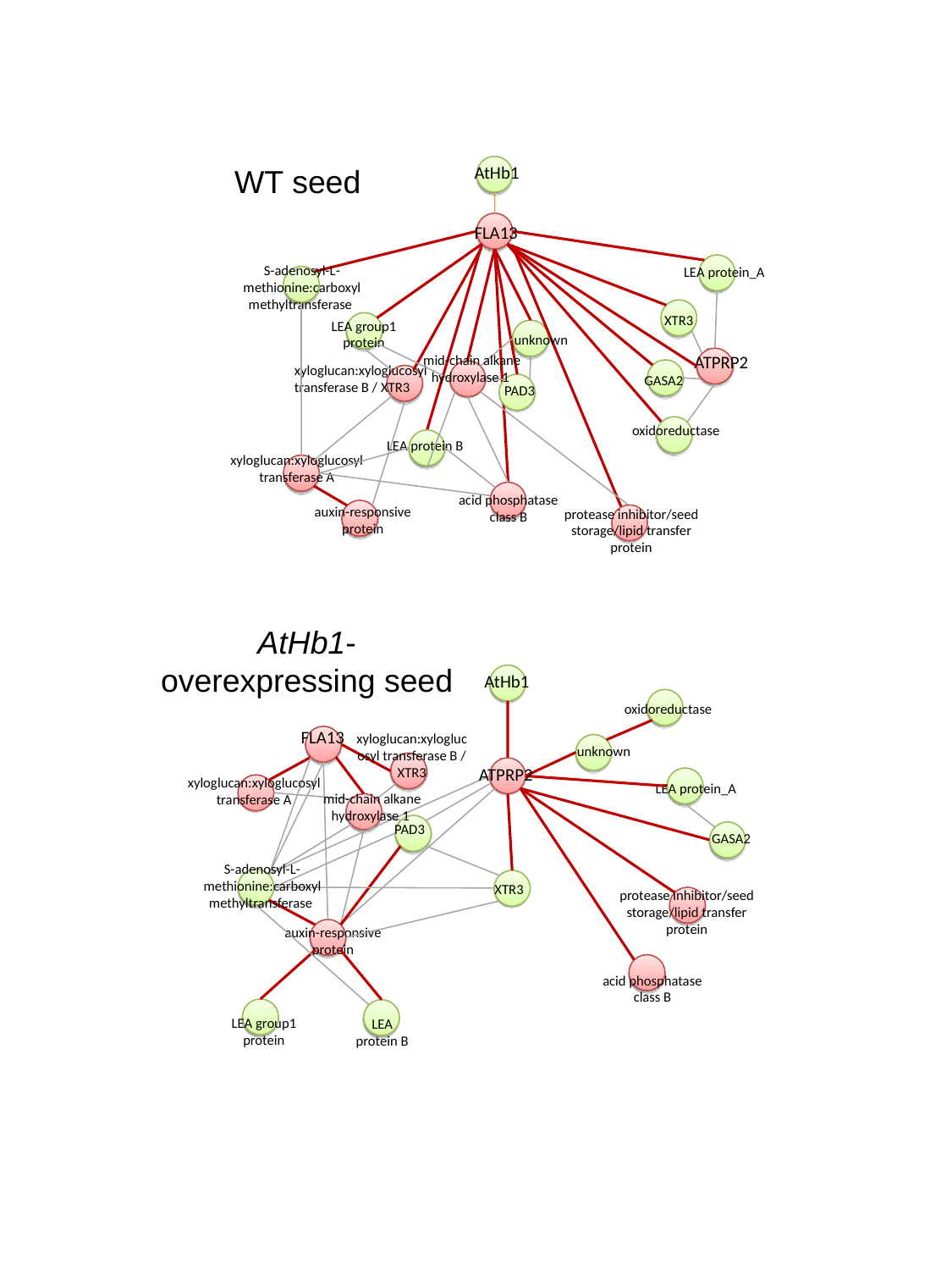

AtHb1
WT seed
FLA13
S-adenosyl-L-methionine:carboxyl methyltransferase
LEA protein_A
XTR3
LEA group1 protein
unknown
AT2G21140
mid-chain alkane hydroxylase 1
ATPRP2
xyloglucan:xyloglucosyl transferase B / XTR3
GASA2
PAD3
oxidoreductase
LEA protein B
xyloglucan:xyloglucosyl transferase A
acid phosphatase class B
auxin-responsive protein
protease inhibitor/seed storage/lipid transfer protein
AtHb1- overexpressing seed
AtHb1
oxidoreductase
FLA13
xyloglucan:xyloglucosyl transferase B / XTR3
unknown
AT2G21140
ATPRP2
xyloglucan:xyloglucosyl transferase A
LEA protein_A
mid-chain alkane hydroxylase 1
PAD3
GASA2
S-adenosyl-L-methionine:carboxyl methyltransferase
XTR3
protease inhibitor/seed storage/lipid transfer protein
auxin-responsive protein
acid phosphatase class B
LEA group1 protein
LEA protein B
